# Recommendations for the content and conduct of European League Against Rheumatism (EULAR) musculoskeletal ultrasound courses

**DOI:** 10.1136/ard.2007.082560

**Published:** 2007-10-05

**Authors:** E Naredo, J W J Bijlsma, P G Conaghan, C Acebes, P Balint, H Berner-Hammer, G A W Bruyn, P Collado, M A D’Agostino, J J de Agustin, E de Miguel, E Filippucci, W Grassi, A Iagnocco, D Kane, J M Koski, B Manger, L Mayordomo, I Möller, C Moragues, E Rejón, M Szkudlarek, L Terslev, J Uson, R J Wakefield, W A Schmidt

**Affiliations:** 1 Rheumatology Department, Hospital Severo Ochoa, Madrid, Spain; 2 Rheumatology & Clinical Immunology Department, University Medical Center, Utrecht, The Netherlands; 3 Academic Section of Musculoskeletal Disease, University of Leeds, Leeds, UK; 4 Rheumatology Department, Fundación Jiménez Díaz, Madrid, Spain; 5 Third General and Paediatric Rheumatology Department, National Institute of Rheumatology and Physiotherapy, Budapest, Hungary; 6 Rheumatology Department, Diakonhjemmet Hospital, Oslo, Norway; 7 Rheumatology Department, Medisch Centrum, Leeuwarden, The Netherlands; 8 Rheumatology Department, Hospital Severo Ochoa, Madrid, Spain; 9 Rheumatology Department, Ambroise Paré Hospital, UVSQ University, Boulogne Billancourt, France; 10 Rheumatology Department, Hospital Vall d’Hebron, Barcelona, Spain; 11 Rheumatology Department, Hospital La Paz, Madrid, Spain; 12 Rheumatology Department, Universitá Politecnica delle Marche, Ancona, Italy; 13 Rheumatology Department, Sapienza University, Rome, Italy; 14 Rheumatology Department, Adelaide and Meath Hospital, Tallaght, Dublin, Ireland; 15 Rheumatology Department, Mikkeli Central Hospital, Mikkeli, Finland; 16 Third Medical Department, University of Erlangen-Nürnberg, Germany; 17 Rheumatology Department, Hospital de Valme, Sevilla, Spain; 18 Rheumatology Department, Instituto Poal, Barcelona, Spain; 19 Rheumatology Department, Hospital de Bellvitge, Barcelona, Spain; 20 Rheumatology Department, University of Copenhagen, Hvidovre Hospital, Copenhagen, Denmark; 21 Rheumatology Department, The Parker Institute, Frederiksberg Hospital, Frederiksberg, Denmark; 22 Rheumatology Department, Hospital de Mostoles, Madrid, Spain; 23 Medical Centre for Rheumatology Berlin-Buch, Berlin, Germany

## Abstract

**Objective:**

To develop education guidelines for the conduct of future European League Against Rheumatism (EULAR) musculoskeletal ultrasound (MSUS) courses.

**Methods:**

We undertook a consensus-based, iterative process using two consecutive questionnaires sent to 29 senior ultrasonographer rheumatologists who comprised the faculty of the 14th EULAR ultrasound course (June 2007). The first questionnaire encompassed the following issues: type of MSUS educational model; course timing; course curriculum; course duration; number of participants per teacher in practical sessions; time spent on hands-on sessions; and the requirements and/or restrictions for attendance at the courses. The second questionnaire consisted of questions related to areas where consensus had not been achieved in the first questionnaire, and to the topics and pathologies to be assigned to different educational levels.

**Results:**

The response rate was 82.7% from the first questionnaire and 87.5% from the second questionnaire. The respondents were from 11 European countries. The group consensus on guidelines and curriculum was for a three-level education model (basic, intermediate and advanced) with timing and location related to the annual EULAR Congresses. The topics and pathologies to be included in each course were agreed. The course duration will be 20 h. There will be a maximum of six participants per teacher and 50–60% of total time will be spent on practical sessions. There was also agreement on prerequisite experience before attending the intermediate and advanced courses.

**Conclusion:**

We have developed European agreed guidelines for the content and conduct of EULAR ultrasound courses, which may also be recommended to national and local MSUS training programmes.

Within the last decade, an increasing number of rheumatologists worldwide have incorporated musculoskeletal ultrasound (MSUS) as a valuable imaging and research tool in their clinical practice.

The European League Against Rheumatism (EULAR) has supported 14 courses on MSUS since 1998. There have been introductory and advanced MSUS courses in different European countries under the auspices of the EULAR Standing Committee on Education and Training. European rheumatologists highly experienced in MSUS have comprised the faculty of these courses. Many of them chair and organise MSUS training for rheumatologists in their own countries.

In 2001, the first guidelines for performing MSUS in rheumatology were published by the EULAR Working Group for Musculoskeletal Ultrasound.^1^ These guidelines provided useful information on the technical basis for MSUS, equipment specifications, scanning methods and image acquisition along with the main pathological findings in each anatomical area.

Within the last 3 years, a number of relevant papers on MSUS education, curriculum and competency for rheumatologists have been published.^2^^–^7 Brown *et al* produced an international interdisciplinary consensus on the specific indications, anatomic areas and knowledge and skills required by rheumatologists performing MSUS.^4^ Nevertheless, there is a wide variety of approaches to training in MSUS.^8^ Furthermore, there is a need for a standardised educational programme of efficient teaching and learning of MSUS for rheumatologists.

The purpose of this project was to develop education guidelines for future EULAR ultrasound courses. These could also be recommended to national and local MSUS training programmes as a formal educational model and curriculum for rheumatologists.

## METHODS

### Study design

The initial step involved a 2-month email-based forum on preliminary ideas for developing MSUS training guidelines, using a core group composed of the organisers of the recent past and future EULAR ultrasound courses. This preliminary approach included discussion on the following issues: (1) the desired model of MSUS education, (2) the theoretical and practical curriculum, (3) the proposed course schedule and duration and (4) the requirements and restrictions for attendance at the courses.

We then undertook a consensus process through two consecutive written questionnaires sent to the 29 senior ultrasonographer rheumatologists who comprised the faculty of the 14th EULAR ultrasound course that was held in Sitges, Spain, 10–13 June 2007. These faculty members were from 12 European countries (Denmark, 2; Finland, 1; France, 2; Germany, 4; Hungary, 1; Ireland, 1; Italy, 3; The Netherlands, 2; Norway, 1; Spain, 10; Switzerland, 1; UK, 1).

### Questionnaire design and content

We developed the first questionnaire based on the issues discussed among the core group. This questionnaire was sent to the 29 rheumatologists who participated as faculty in the 14th EULAR ultrasound course, and they were asked to respond within 2 months. An explanation of the purpose of the exercise accompanied the questionnaire. After 4 weeks, e-mail reminders were sent to the non-responders.

The first questionnaire included 30 questions divided into 8 sections to be answered by ticking “yes” or “no” with space for additional comments. Participants were allowed to vote on more than one option for each section. The eight sections that comprised the first questionnaire included the following topics: MSUS educational model, course timing, course curriculum, course duration, number of participants per teacher in practical sessions, time spent on hands-on sessions, requirements, and/or restrictions for attendance at the courses. The educational model section offered four possibilities: two-level education, three-level education (eg basic, intermediate and advanced), two-level education and additional courses on selected advanced subjects, and two-level education and additional modular courses on specific anatomic areas and/or diseases or group of diseases. The course timing section offered four possibilities: ultrasound courses with timing and location closely related to the annual EULAR Congresses, ultrasound courses scheduled apart from the annual EULAR Congresses, the basic or the basic and intermediate courses with timing and location closely related to the annual EULAR Congresses and the advanced course at different time. The course curriculum section offered four questions about the main educational objectives of the courses (eg examination technique versus examination technique plus basic pathology for the basic course; MSUS research in rheumatology versus pathological findings in other specialities for the advanced course). The course duration section provided three possibilities: 20 h over 3 days, 24 h over 3 days, and 20 h over 2.5 days. With regard to the number of participants per teacher in practical sessions, three options (four, five or six participants) were given. The offered possibilities of time spent on “hands-on” sessions were 40–50%, 50–60% or 60–70% of the total duration of each course. Nine questions about different possibilities of requirements and/or restrictions for attendance at the courses were included in the last section.

The second questionnaire consisted of two parts. Firstly, seven questions were related to areas of non-consensus and comments supplied in the first questionnaire, to be answered by ticking “yes” or “no” with space for any additional comments. Secondly, it included a list of 22 topics and 29 MSUS pathologies to be assigned to different levels of education, together with space for any suggestions of other topics or pathologies not included in the provided list. Participants were allowed to vote on more than one option for each section. The second questionnaire and the results from the first questionnaire were sent by e-mail to the responders to the first questionnaire. They were asked to respond within 2 months and after 4 weeks, e-mail reminders were sent to the non-responders.

### Analysis

We calculated the percentage of respondents who answered yes and no to the questions included in each questionnaire. Group agreement with the issue under consideration was defined as agreement ⩾65%.

## RESULTS

The response rate was 82.7% (24 out of 29) from the first questionnaire and 87.5% (21 out of 24) from the second questionnaire. The respondents were from 11 European countries (Denmark, 2; Finland, 1; France, 1; Germany, 2; Hungary, 1; Ireland, 1; Italy, 3; The Netherlands, 1; Norway, 1; Spain, 10; UK, 1). Because of the high percentage of respondents from one European country (Spain) with respect to the other countries, we calculated again the results for each question taking into account the answers from a maximum of four Spanish ultrasonographer rheumatologists. The four Spanish rheumatologists who had comprised the faculty of more EULAR ultrasound courses than the others were selected for this second analysis. The results of this separate analysis are only reported when they were different from the total group.

### Results from the first questionnaire round

The total agreement for a three-level education model was 65.2%. A total of 17.4% of the respondents voted for a two-level education model, 30.4% voted for two-level education and additional courses on selected advanced subjects and 26.1% voted for two-level education and additional modular courses on specific anatomic areas and/or diseases or group of diseases. Eight (33.3%) respondents voted for two options. After analysing again the answers including only four Spanish respondents, 50% of the respondents voted for a three-level education model, 22.2% of the respondents voted for a two-level education model, 33.3% voted for two-level education and additional courses on selected advanced subjects and 22.2% voted for two-level education and additional modular courses.

Ultrasound courses with timing and location closely related to the annual EULAR Congresses was agreed by 87% of the respondents. However, there were comments on the possibility that future EULAR Congresses may take place in cities/countries where there are no colleagues willing or able to organise the MSUS courses.

In all, 48.3% of the respondents voted that the basic course should include only ultrasound examination technique and 48.3% that it should also include basic pathological findings. A total of 74% of the respondents voted that advanced course should include MSUS research and methodology, an update on MSUS in rheumatology and technological developments, and 65.2% of the respondents voted that the advanced course should include MSUS uncommon findings in rheumatology as well as MSUS findings in other specialities.

The agreement on course duration was 48.3% for 20 h over 3 days, 43.5% on 24 h over 3 days and 13% on 20 h over 2.5 days.

A maximum of six participants per teacher, and ideally four or five participants per teacher, was voted by 100% of respondents.

With regard to time spent on “hands-on” scanning, more than 65% of the respondents voted for 50–60% of the total duration of each course.

In all, 61% of respondents agreed that there should be some restrictions to participating in EULAR ultrasound courses. Only 8.7% voted for restrictions in attending the basic course, 69.6% for restricting attendance at the intermediate course and 82.6% voted for restrictions on attending the advanced course. After considering only four Spanish respondents, 44.4% voted for some restrictions to participating in EULAR ultrasound courses, 5.5% for restrictions in attending the basic course, 61.1% for restricting attendance at the intermediate course and 72.2% voted for restricting attendance at the advanced course.

### Results from the second questionnaire round

A proposal was made for three-level courses with timing and location closely related to the annual EULAR Congress if local organisers are willing and able to conduct such courses; as well, if future EULAR Congresses take place in cities/countries where there are no colleagues willing or able to organise the MSUS courses, the courses would be organised by another colleague in their own country/city. This was agreed by 95.2% of the respondents.

In all, 95.2% of the respondents voted that the basic course should focus on ultrasound examination techniques and basic pathology, the intermediate course should focus on a wide spectrum of rheumatologic pathology, and the advanced course should focus on current MSUS research in rheumatology, new technological developments, uncommon pathological findings in rheumatology, pathological findings in other specialities (such as nerve, ligament, muscle and sport-related lesions), and MSUS methodology.

The total agreement for course duration of 20 h for 3 days was 70%.

The proposal was overwhelmingly agreed that there will be no restrictions on attending basic courses: however, the proposals that previous performance of ⩾100 MSUS scans before participating in an intermediate level course, and previous performance of ⩾300 MSUS scans before attending an advanced level course were agreed by 90.5% of the respondents.

Eight topics and five pathologies were assigned to the basic course by more than 70% of the respondents, 7 topics and 16 pathologies to the intermediate course and 8 topics and 9 pathologies to the advanced course (table 1).

**Table 1:** Musculoskeletal ultrasound (MSUS) course curriculum and pathology content for basic, intermediate and advanced levels

Level	Content
Basic	Application, indications and limitations of MSUS in rheumatology.
	Ultrasound physics and technology
	Sonographic pattern of the different musculoskeletal tissues
	MSUS artefacts and pitfalls
	Standard sonographic scans of the shoulder, elbow, wrist and hand, hip, knee, ankle and foot
	Holding the probe and optimising the grey-scale settings of the sonographic system
	Image documentation
	Reporting ultrasound findings and diagnosis
	Basic course pathologies
	Joint synovitis
	Joint effusion
	Synovial hypertrophy
	Bursitis
	Tenosynovitis
	
Intermediate	Colour and power Doppler physics and technology
	Application, indications and limitations of colour and power Doppler in rheumatology
	Use of the colour and power Doppler settings
	Colour and power Doppler artefacts
	Use of colour and power Doppler to detect synovial and entheseal inflammation
	Assessment and quantification of structural joint damage (bone, tendons, ligaments)
	Sonographic-guided periarticular and articular injections
	Intermediate course pathologies
	Joint synovitis, synovial hypertrophy, tenosynovitis
	Tendon calcification
	Enthesopathy
	Tendinosis
	Paratenonitis
	Tendon subluxation/luxation
	Intrasubstance tendon lesions
	Tendon impingement
	Complete tendon tear
	Partial tendon tear
	Bone erosions
	Osteophytes
	Ganglia and cysts
	Articular cartilage lesions
	Peri- and intra-articular microcrystal deposit
	Ligament, muscle, cartilage, fibrocartilage and synovial calcification
	
Advanced	Optimisation of colour and power Doppler settings
	Sonographic-guided musculoskeletal interventional procedures
	Assessment and quantification of synovial, tenosynovial and entheseal inflammatory activity
	Role of ultrasound in vasculitis
	Evaluation of vessels and detection of vasculitis by sonography
	Paediatric sonography: musculoskeletal sonoanatomy and pathological findings in rheumatic diseases
	Uncommon sonographic pathological findings in rheumatology
	MSUS technological development
	3- and 4-dimensional MSUS
	Update on MSUS in rheumatology
	MSUS research and methodology
	Advanced course pathologies
	Peripheral nerve entrapment and lesions
	Ligament lesions
	Fibrocartilage lesions
	Myopathy
	Myositis
	Muscle injury
	Soft tissue masses
	Loose bodies
	Foreign bodies

In summary, the group consensus on guidelines and curriculum for future EULAR MSUS courses are outlined below.

#### 1. MSUS education model

A three-level education model with basic, intermediate and advanced levels will be conducted.

#### 2. Course timing

There will be three-level courses with timing and location related to the annual EULAR Congresses if local organisers are willing and able to conduct the courses. If future EULAR Congresses take place in cities/countries where there are no colleagues willing or able to organise the MSUS courses, another colleague can organise three courses (basic, intermediate and advanced)/simultaneously in their country/city. With this course timing, 2 years is the minimum time in which all three courses can be attended by trainees interested in MSUS. They would have a minimum of 1 year for practising between consecutive levels.

#### 3. Course curriculum

Basic courses will focus on examination technique and will include some basic pathology. Intermediate courses will focus on a wide spectrum of rheumatologic pathology. Advanced courses will focus on current MSUS research in rheumatology, new technological developments, uncommon pathological findings in rheumatology, pathological findings in other specialities such as nerve, ligament, muscle lesions, sport related lesions and MSUS methodology (table 1).

#### 4. Course duration

A total of 20 h for 3 days.

#### 5. Number of participants per teacher in practical sessions

Ideally four or five, maximum six participants per teacher.

#### 6. Time spent on hands-on scanning

In all, 50–60% of total time will be spent in practical training for all courses; 40–50% of total time will be theoretical teaching.

#### 7. Attendance at the EULAR ultrasound courses

There will be no prerequisites for attending basic courses. Previous performance of ⩾100 musculoskeletal ultrasound scans is strongly recommended before participating in an intermediate-level course and previous performance of ⩾300 musculoskeletal ultrasound scans is strongly recommended before attending an advanced-level course.

#### 8. Certification

The group voted for working towards certification of attendance and competency.

## DISCUSSION

High-resolution MSUS has become an established imaging technique for evaluating periarticular and intra-articular structures involved in rheumatic diseases.^9^^–^11 In addition, ultrasound is a bedside tool for performing accurate and safe musculoskeletal injections.^12^

Within the last decade, several reports on the superiority of US over clinical evaluation and plain radiography for assessing early joint inflammatory and structural changes have directed MSUS applications in rheumatology towards early diagnosis, assessment of disease activity and monitoring of therapeutic response in patients with inflammatory arthritis.^13^^–^21

As a consequence of the recent technological development and increasing utility of MSUS, there is a great demand for appropriate education in this technique among rheumatologists worldwide. Since US is an operator-dependent imaging technique (mainly because of the intrinsic real time nature of US images acquisition) appropriate training is highly important to ensure skilled and safe use of MSUS by rheumatologists.^22^ ^23^

Sustained and extensive interest has occurred in attending EULAR ultrasound courses that have been organised over the last 9 years. Furthermore, several MSUS courses and workshops are offered by individual European national Societies of Radiology and Rheumatology and by Universities in many countries. In addition, MSUS is a compulsory part of rheumatology training in some European countries. However, until now there has been no agreed educational programme and curriculum on MSUS for European rheumatologists. We therefore aimed to develop guidelines on content and conducting MSUS courses for rheumatologists under the auspices of EULAR. These guidelines can also be followed by national and local societies and/or universities in order to standardise MSUS training across Europe. Standardisation of the MSUS education model is essential for validating the results of training in this technique.

We used a consensus method among European rheumatologists highly experienced in performing and teaching MSUS. They were asked to vote on different options regarding content and conduct of MSUS courses. Controversial points were voted again in the next round after presenting the group replies from the previous round. The high response rate reflects their great motivation for MSUS education.

Because there was a higher number of respondents from one European country (Spain) than from the other countries, we analysed again the questionnaires taking into account only the answers from the four Spanish ultrasonographer rheumatologists who had participated as faculty in more EULAR ultrasound courses than their peers in order to avoid unbalanced influence from the country in question. After excluding the answers from six Spanish rheumatologists, the results did not change considerably.

The most controversial issue concerned how many levels of MSUS training should be included. Arguments for the three-level education model (basic, intermediate and advanced) included that this would result in a higher competency achieved by trainees, more homogeneous groups in practical sessions in each course level, increased time for practical sessions in each course and a step-by-step training in MSUS. However, three-level courses may be more difficult to organise and conduct, as well as more time consuming for participants and teachers than two-level courses. Nevertheless, the 14th EULAR ultrasound course consisted of three simultaneous basic, intermediate and advanced courses (10–13 June 2007, Sitges, Spain). This course was successfully carried out with a careful, pre-planned organisation.

With regard to the other educational models proposed, they were more frequently voted for than the two-level education model. The two-level education model plus additional courses on selected advanced subjects is quite similar to a three-level education model. The two-level education model plus modular courses on anatomic areas and/or diseases would lead to a higher number of courses than in the three-level model, increasing time and money spent on ultrasound education by trainees and trainers.

The total agreement for a three-level education model decreased from 65.2% to 50% after considering only four Spanish respondents. The six excluded Spanish rheumatologists voted for a three-level education model because most were involved as organisers of the 14th EULAR ultrasound course. Nevertheless, a three-level education model was agreed on by more respondents than a two-level education model (22%), two-level education and additional courses on selected advanced subjects (33%) and two-level education and additional modular courses (22%).

With the three-level course timing, trainees can attend a course each year. They need a minimum of 2 years for attending basic, intermediate and advanced course and they have a minimum of 1 year for practising between consecutive levels. We consider that 2 years should be the minimal time necessary for achieving competency in MSUS.

For the curriculum of the courses, we selected the principal topics and pathologies that have been considered relevant and appropriate for rheumatologic practice according to published data.^6^ Afterwards, they were assigned to the basic, intermediate and advanced level according to the results from the second questionnaire.

Since practical training under expert supervision is essential for appropriate MSUS learning, the courses should have a 50% to 60% of total time spent in practical, hands-on sessions and no more than six participants per tutor in such sessions.

Although the courses should be considered as a necessary starting point for developing and improving MSUS skills, training after courses by performing normal scans and diagnostic examinations is mandatory for consolidating the knowledge and skills provided during the courses. Then, a number of ultrasound examinations are recommended before attending intermediate and advanced courses.

Certification is desirable. Guidelines for certification of competency will be developed in cooperation with the EULAR Standing Committee on Education and Training, the EULAR Committee on Musculoskeletal Imaging, and with other European institutions.

In conclusion, we have developed European agreed guidelines for content and conducting EULAR MSUS courses, which will be also useful for standardising rheumatology MSUS training worldwide.
